# Association between workplace bullying and burnout, professional quality of life, and turnover intention among clinical nurses

**DOI:** 10.1371/journal.pone.0226506

**Published:** 2019-12-20

**Authors:** Yujeong Kim, Eunmi Lee, Haeyoung Lee

**Affiliations:** 1 College of Nursing, Kyungpook National University, Daegu, Republic of Korea; 2 Department of Nursing, Hoseo University, Asan, Republic of Korea; 3 College of Nursing, Chung-Ang University Red Cross, Seoul, Republic of Korea; Victoria University, AUSTRALIA

## Abstract

Workplace bullying experienced by clinical nurses is associated with burnout, a factor that threatens the quality of nursing care and patient safety. This study examined the association of workplace bullying with burnout, professional quality of life, and turnover intention among clinical nurses. A descriptive cross-sectional study was conducted using a structured questionnaire. Data were collected from 324 nurses and were analyzed using t-test, one-way analysis of variance, and multiple regression. Controlling for the general characteristics of the participants, workplace bullying had a significant association with emotional exhaustion (B = 0.29, p < 0.01) and depersonalization (B = 0.15, p < 0.01) among the subdomains of burnout, compassion fatigue among the components of professional quality of life (B = 0.15, p < 0.01), and turnover intention (B = 0.05, p < 0.01). Thus, preventing workplace bullying is important to reduce clinical nurses’ burnout and turnover. The role of nursing leadership is crucial to develop interventions that reduce workplace bullying and successfully create a professional, nurturing, and supportive work culture.

## Introduction

Workplace bullying has been a serious social issue since the early 2000s, with numerous studies conducted on the subject. Bullying is more frequently reported among nurses than persons in other occupations [1,2]. Workplace bullying experienced by nurses can take various forms, including personal bullying, job-related bullying, and intimidation-related bullying [3,4]. The negative outcomes of workplace bullying vary from reduced self-esteem [5] to suicide [6].

Constant exposure to stressful situations caused by workplace bullying is associated with an increased risk of hypertension and heart disease [7], and may lead not only to physical problems (e.g., physical discomfort, fatigue, and angina) [8,9] but also to mental health issues (e.g., anxiety, depression, and posttraumatic stress disorder) [10]. Moreover, studies have shown that workplace bullying might cause job-related problems such as decline in job satisfaction, productivity reduction, poor job performance, burnout, and increased turnover intention [11,12]. Furthermore, it has an effect not only on first-hand victims but also on those who witness the bullying, as they experience similar somatic complaints and psychological disturbances [13,14]. Altogether, workplace bullying increases turnover among nurses and acts as a factor disrupting the advancement of organizations [15]. Additionally, it may also threaten patient safety by lowering nursing care quality; therefore, more awareness and solutions are required to prevent workplace bullying [16,17].

Professional quality of life (ProQoL) refers to the quality of life as perceived by professional workers who provide services to others, and comprises both positive and negative aspects [18]. Compassion satisfaction, a positive aspect of ProQoL, involves the emotional satisfaction experienced by professional service providers when helping their clients; it also includes positive feelings about their colleagues and their own capacity to help others [18]. A negative aspect of ProQoL is compassion fatigue, which includes burnout and secondary trauma (i.e., the trauma caused by work associated with extreme stress) [18]. Low ProQoL is a critical issue among nurses, as it leads to decreased concern toward patients, which can negatively affect treatment outcomes. On the other hand, compassion satisfaction has a decisive function in buffering the negative effects of compassion fatigue, including burnout, and improving overall mental well-being [19,20].

Turnover intention refers to the tendency to switch jobs or change one’s occupation owing to dissatisfaction with work [21], and is a leading variable for turnover. To provide high-quality nursing care for patients, it is important not only to acquire capable nurses but also to establish a work environment that prevents burnout and turnover intention caused by violence and that enhances nurses’ ProQoL; such a working environment may ultimately lead to increased years of service and reduced costs of human resources management.

Hence, this study examined the current status of workplace bullying experienced by clinical nurses and its association with burnout, ProQoL, and turnover intention. In particular, this study considered the various components of workplace bullying, burnout, and ProQoL. This could provide basic data for the strategic development of a positive organizational culture within the nursing community and thus prevent the incidence of bullying experienced by clinical nurses.

## Materials and methods

### Study design

This study used a descriptive cross-sectional design to identify the association of workplace bullying with burnout, ProQoL, and turnover intention among clinical nurses.

### Participants

Study participants were nurses currently employed in general hospitals in Seoul, Gyeonggi, and Chungnam, who directly participated in patient care. Newly hired nurses assigned to a hospital but still not undertaking independent tasks were excluded. The number of participants was determined using G*Power [22]. Considering an effect size of 0.25, significance level of 0.05, and power of 0.95, the total number of participants was 305. To compensate for dropouts, 339 participants were recruited into the study. Among them, three questionnaires with missing values were excluded. From the 336 completed questionnaires, data from 12 participants who reported not having experienced workplace bullying since joining the hospital were also excluded from the study. Finally, data from 324 participants were analyzed in this study. All participants consented to participate in the study upon understanding its objectives and signed the informed consent form.

### Measurements

The structured questionnaire used in the study contained a total of 84 questions: 6 on general characteristics, 22 on workplace bullying, 22 on burnout, 30 on ProQoL, and 4 on turnover intention.

#### General characteristics

The characteristics of the participants included gender, years of experience, position, work schedule, and job satisfaction.

#### Workplace bullying

To assess workplace bullying, Einarsen, Hoel, and Notelaers [23] developed and validated the Negative Acts Questionnaire-Revised (NAQ-R), a revised English version of the Negative Acts Questionnaire. In our study, we used the Korean version of the NAQ-R translated by Nam, Kim, Kim, Koo, and Park [24], with verified reliability and validity. The NAQ-R consists of 22 questions: 12 on personal bullying, 7 on work-related bullying, and 3 on intimidation-related bullying. Each item is evaluated on a 5-point scale according to the respondent’s frequency of bullying experiences within the last 6 months. Scores range from 22 to 110, with higher scores indicating a higher level of bullying. Cronbach’s α was 0.93 when the tool was developed [24]; in this study, it was 0.95.

#### Burnout

Burnout was examined using the Maslach Burnout Inventory (MBI) [25], translated into Korean and validated by Kang and Kim [26]. The MBI comprises three subdomains with a total of 22 items: emotional exhaustion (9 items), depersonalization (5 items), and decreased personal accomplishment (8 items). Each question is evaluated on a 7-point scale (0–6 points). The MBI (published by Mind Garden, Inc., www.mindgarden.com) is a tool with high reliability and validity to assess burnout in personal service occupational groups, including nurses [27]. In Kang and Kim’s [26] validation study, Cronbach’s α was 0.85 (overall scale), 0.91 (emotional exhaustion), 0.79 (depersonalization), and 0.84 (decreased personal accomplishment). In the present study, it was 0.87 (overall scale), 0.91 (emotional exhaustion), 0.78 (depersonalization), and 0.84 (decreased personal accomplishment).

#### ProQoL

ProQoL was measured with the ProQoL tool developed by Stamm [18]; we used the Korean version translated by Bae and Lee [28]. The tool comprises two subscales: compassion satisfaction (positive) and compassion fatigue (negative). Burnout and secondary traumatic stress are components of compassion fatigue. In this study, we assessed compassion fatigue using only the items of secondary traumatic stress and excluding burnout. The tool comprises 10 items for each domain, rated on a 5-point scale; the range of subdomain scores is 10–50. In this study, Cronbach’s α for compassion satisfaction and secondary traumatic stress was 0.88 and 0.84, respectively.

#### Turnover intention

Turnover intention was assessed with the four questions developed by Lawler [21]; we used the version revised by Park [29] to be used with nurses. The tool comprises four questions, each rated on a 5-point Likert-type scale ranging from 1 (not at all) to 5 (very much so). Higher scores indicate a greater turnover intention. Cronbach’s α was 0.88 in Park’s study [29], and 0.77 in the present study.

### Data collection

Data were collected between July 1, 2018 and September 30, 2018, after obtaining approval from the Institutional Review Board of the researchers’ university. Structured self-administered questionnaires were used for data collection. The researchers visited the hospitals and posted announcements for participation in the study. The questionnaires were distributed to and collected from the nurses who agreed to participate. The participant nurses were briefed about the study objectives, confidentiality of data, anonymity, and freedom to refuse or cease participation at will. The questionnaire was completed only once. Nurses spent approximately 30 minutes listening to the study objectives and completing the questionnaire. Coupons for drinks were given to the participant nurses.

### Statistical analysis

Data were analyzed using SPSS 20.0 (IBM Corp., Armonk, NY, USA). Participants’ general characteristics, workplace bullying, burnout, ProQoL, and turnover intention were analyzed using frequencies, percentages, means, and standard deviations. Differences in workplace bullying, burnout, ProQoL, and turnover intention according to participants’ characteristics were analyzed using t-tests and one-way analysis of variance. Correlations between participants’ workplace bullying, burnout, ProQoL, and turnover intention were analyzed with Pearson correlation coefficient. The associations of participants’ characteristics and workplace bullying with burnout, ProQoL, and turnover intention were analyzed using multiple regression.

## Results

### Characteristics of participants

A total of 324 nurses participated in this study, including 312 females (96.3%) and 12 males (3.7%). Regarding years of experience, 124 participants (38.3%) had worked for less than 1.9 years, accounting for the largest group, and 88 (27.1%) had worked 2–4.9 years; in total, the average was 5.4 years of experience. Regarding position, 301 (92.9%) and 23 (7.1%) participants were staff nurses and charge nurses, respectively. As for employment type, 85 participants (26.2%) had full-time employment, and 239 (73.8%) had rotational shiftwork. Regarding job satisfaction, 69 participants (21.3%) reported being satisfied, 161 (49.7%) were neutral, and 94 (29.0%) were dissatisfied (Table 1).

**Table 1:** Characteristics of participants (n = 324).

Characteristics	Categories	n (%)
Gender	Female	312 (96.3)
	Male	12 (3.7)
Years of experience	≤1.9	124 (38.3)
	2–4.9	88 (27.1)
	5–9.9	43 (13.3)
	≥10	69 (21.3)
Position	Staff nurse	301 (92.9)
	Charge nurse	23 (7.1)
Work schedule	Day fixed	85 (26.2)
	Rotating shift	239 (73.8)
Job satisfaction	Satisfied	69 (21.3)
	Neutral	161 (49.7)
	Dissatisfied	94 (29.0)

### Workplace bullying, burnout, ProQoL, and turnover intention

Regarding workplace bullying, the mean scores ± standard deviations for personal, work-related, and intimidation-related bullying were 22.23 ± 10.75, 10.06 ± 3.97, and 7.11 ± 3.12, respectively. Regarding burnout, the mean scores for emotional exhaustion, depersonalization, and decreased personal achievement were 30.77 ± 11.26, 13.31 ± 6.52, and 18.29 ± 8.04, respectively. Regarding ProQoL, the mean scores for compassion satisfaction and compassion fatigue (secondary traumatic stress) were 29.38 ± 6.18 and 28.66 ± 6.40, respectively. The mean score for turnover intention was 13.12 ± 3.63 (Table 2).

**Table 2:** Descriptive statistics for workplace bullying, burnout, ProQoL, and turnover intention (n = 324).

	Variables	Mean	SD	Range
Workplace bullying		39.40	16.53	22–110
	Personal bullying	22.23	10.75	12–60
	Work-related bullying	10.06	3.97	5–25
	Intimidation-related bullying	7.11	3.12	5–25
Burnout		62.37	18.58	0–132
	Emotional exhaustion	30.77	11.26	0–54
	Depersonalization	13.31	6.52	0–30
	Decreased personal achievement	18.29	8.04	0–48
ProQoL		58.04	8.10	20–100
	Compassion satisfaction	29.38	6.18	10–50
	Compassion fatigue	28.66	6.40	10–50
Turnover intention		13.12	3.63	4–20

ProQoL: professional quality of life, SD: standard deviation.

### Differences in workplace bullying, burnout, ProQoL, and turnover intention by general characteristics

Workplace bullying differed significantly according to years of experience, type of employment, and job satisfaction. In particular, personal, work-related, and intimidation-related bullying all differed significantly based on the type of employment and job satisfaction. Personal and work-related bullying also differed according to years of experience. Compared with nurses with 10 or more years of experience, those who had less than 5 years of experience scored higher on personal and work-related bullying.

Burnout differed significantly according to years of experience, position, type of employment, and job satisfaction. Emotional exhaustion and depersonalization differed significantly according to all descriptive characteristics except for gender. Decreased personal achievement showed significant differences according to position, type of employment, and job satisfaction. Nurses with less than 5 years of experience scored significantly higher in burnout than nurses with 10 years of experience or more. Burnout scores were significantly higher in staff nurses than in charge nurses, in nurses with rotational shiftwork than those with full-time employment, and in nurses dissatisfied with work than those satisfied with work.

Regarding ProQoL, compassion satisfaction differed significantly according to all variables except for gender. Compassion fatigue differed significantly according to years of experience, type of employment, and job satisfaction. Nurses who had less than 5 years of experience showed significantly lower compassion satisfaction and higher compassion fatigue than nurses who had 10 or more years of experience. Nurses who had rotational shiftwork showed significantly lower compassion satisfaction and higher compassion fatigue than did full-time nurses. Job satisfaction showed similar results.

There were significant differences in nurses’ turnover intention with respect to their years of experience, position, type of employment, and job satisfaction. Turnover intention was significantly higher in nurses who had less than 5 years of experience compared to nurses who had 10 or more years of experience, staff nurses than charge nurses, nurses with rotational shiftwork than those with full-time employment, and dissatisfied nurses than satisfied or neutral nurses.

All the results regarding the differences in workplace bullying, burnout, ProQoL, and turnover intention by general characteristics are shown in Table 3.

**Table 3:** Differences in workplace bullying, burnout, ProQoL, and turnover intention by general characteristics (n = 324).

Characteristics				Workplace bullying								Burnout									ProQoL						Turnover intention		
		Personal				Work-related			Intimidation-related			Emotional exhaustion			Depersonalization			Decreased personal achievement			Compassion satisfaction			Compassion fatigue					
		Mean	SD		t or F (p)	Mean	SD	t or F (p)	Mean	SD	t or F (p)	Mean	SD	t or F (p)	Mean	SD	t or F (p)	Mean	SD	t or F (p)	Mean	SD	t or F (p)	Mean	SD	t or F (p)	Mean	SD	t or F (p)
Sex																													
	Female	22.24	10.84		0.13	10.05	3.95	-0.24	7.11	3.12	-0.07	30.88	11.32	0.92	13.38	6.52	1.07	18.28	8.05	-0.13	29.30	6.11	-1.12	28.76	6.43	0.24	13.15	3.65	0.61
	Male	21.83	8.39		(0.90)	10.3±	4.70	(0.81)	7.17	3.07	(0.95)	27.83	9.72	(0.36)	11.33	6.64	(0.29)	18.58	8.04	(0.90)	31.33	7.83	(0.27)	26.17	4.93	(0.17)	12.50	3.45	(0.55)
Years of experience																													
	≤1.9^a^	25.60	12.38		10.35	10.65	4.08	5.1	7.59	3.57	2.68	35.30	11.18	16.14	14.85	6.62	6.95	19.35	7.86	2.33	28.14	6.37	5.13	30.61	6.92	8.88	13.75	3.89	8.19
	2–4.9^b^	22.67	10.18		(<0.01)	10.67	4.26	(<0.01)	7.25	2.87	(<0.05)	30.28	11.12	(<0.01)	13.64	6.43	(<0.01)	18.66	7.70	(0.07)	29.10	5.22	(<0.01)	28.52	5.59	(<0.01)	13.81	2.92	(<0.01)
	5–9.9^c^	18.65	7.16		a>c,d	9.37	3.27	a,b>d	6.49	2.35	a>c,d	28.53	8.39	a>b,c,d	12.53	5.80	a,b>d	17.72	6.46		29.88	5.33	a,b<d	27.63	5.83	a>c,d	12.70	2.85	a,b<d
	≥10^d^	17.84	7.67		b>d	8.65	3.41		6.45	2.81		24.62	9.79	b>d	10.59	6.05		16.28	9.34		31.65	6.87		25.99	5.61		11.39	3.89	
Position																													
	Staff nurse	22.53	10.93		1.86	10.11	3.98	0.78	7.17	3.17	1.215	31.15	11.32	2.23	13.58	6.52	2.75	18.54	7.91	2.05	29.00	6.04	-4.06	28.68	6.39	0.18	13.25	3.54	2.33
	Charge nurse	18.22	7.03		(0.06)	9.43	3.93	(0.44)	6.35	2.25	(0.23)	25.74	9.28	(0.03)	9.74	5.45	(0.01)	15.00	9.15	(0.04)	34.30	5.93	(<0.01)	28.43	6.62	(0.86)	11.43	4.47	(0.02)
Work schedules																													
	Day fixed	18.82	9.07		-3.79	8.65	3.40	-4.23	6.01	1.94	-4.92	25.66	11.32	-5.05	11.04	6.38	-3.820	16.09	8.08	-2.97	31.46	6.46	3.68	26.76	6.38	-3.24	12.26	3.73	-2.58
	Rotating shift	23.44	11.06		(<0.01)	10.56	4.04	(<0.01)	7.50	3.36	(<0.01)	32.58	10.69	(<0.01)	14.12	6.39	(<0.01)	19.08	7.90	(<0.01)	28.64	5.92	(<0.01)	29.34	6.27	(<0.01)	13.43	3.56	(0.01)
Job satisfaction																													
	Satisfied	18.46	8.50		22.33	8.35	3.09	19.98	6.26	2.72	14.78	21.99	8.80	56.84	9.29	5.61	31.47	14.57	7.79	10.79	34.23	5.61	44.88	25.43	5.60	32.15	10.13	3.62	65.04
	Neutral	20.49	9.23		(<0.01)	9.68	3.59	(<0.01)	6.66	2.58	(<0.01)	30.13	10.16	(<0.01)	13.01	6.04	(<0.01)	18.83	8.19	(<0.01)	29.27	5.51	(<0.01)	27.82	6.10	(<0.01)	12.94	3.02	(<0.01)
	Dissatisfied	27.97	12.37		a,b<c	11.9±	4.42	a,b<c	8.50	3.74	a,b<c	38.30	9.55	a<b<c	16.77	6.14	a<b<c	20.11	7.11	a<b,c	26.01	5.32	a<b,c b>c	32.48	5.60	a,b<c	15.64	2.75	a<b<c

ProQoL: professional quality of life; SD: standard deviation.

### Correlations of workplace bullying, burnout, ProQoL, and turnover intention

The subdomains of workplace bullying showed significant correlations with burnout (except decreased personal achievement), ProQoL, and turnover intention. No significant correlation was observed for decreased personal achievement with personal bullying, work-related bullying, emotional exhaustion, or depersonalization. Positive correlations were observed for workplace bullying with burnout, compassion fatigue, and turnover intention (Table 4).

**Table 4:** Correlations between workplace bullying, burnout, professional quality of life, and turnover intention (n = 324).

		Workplace bullying			Burnout			ProQoL		Turnover intention
		Personal	Work-related	Intimidation-related	Emotional exhaustion	Depersonalization	Decreased personal achievement	Compassion satisfaction	Compassion fatigue	
Workplace bullying	Personal	1								
	Work-related	0.748**	1							
	Intimidation-related	0.768**	0.681**	1						
Burnout	Emotional exhaustion	0.568**	0.554**	0.439**	1					
	Depersonalization	0.459**	0.470**	0.427**	0.695**	1				
	Decreased personal achievement	0.108	0.088	0.115*	0.002	0.082	1			
ProQoL	Compassion satisfaction	-0.172**	-0.115*	-0.117*	-0.314**	-.365**	-0.578**	1		
	Compassion fatigue	0.515**	0.448**	0.384**	0.671**	0.533**	0.124*	-0.169**	1	
Turnover intention		0.384**	0.355**	0.289**	0.487**	0.403**	0.169**	-0.427**	0.399**	1

ProQoL: professional quality of life.

* p < 0.05

** p < 0.01

### Associations of general characteristics and bullying with burnout, professional quality of life, and turnover

According to the results of the multiple regression analysis conducted to examine the associations of workplace bullying with burnout, ProQoL, and turnover intention, when the general characteristics of participants were controlled for, workplace bullying had a significant effect on emotional exhaustion and depersonalization among the subdomains of burnout, as well as on compassion fatigue and turnover intention (Table 5).

**Table 5:** Associations of general characteristics and bullying with burnout, professional quality of life, and turnover intention (n = 324).

General characteristics		Burnout						ProQoL				Turnover intention	
		Emotional exhaustion		Depersonalization		Decreased personal achievement		Compassion satisfaction		Compassion fatigue			
		B	p	B	p	B	p	B	p	B	p	B	p
Years of experience	≤1.9 vs. ≥10	5.34	<0.01	1.53	0.02	0.09	0.10	-1.58	0.01	1.74	<0.01	0.07	0.13
	2–4.9 vs. ≥10	0.01	0.86	0.03	0.60	-0.02	0.74	-0.02	0.78	0.01	0.93	0.05	0.32
	5–9.9 vs. ≥10	0.02	0.68	0.01	0.84	-0.03	0.55	0.01	0.87	0.02	0.76	-0.05	0.33
Position	Staff nurse vs. charge nurse	0.01	0.80	0.08	0.10	0.08	0.16	-3.64	<0.01	-0.09	0.06	0.06	0.20
Work schedule	Day fixed vs. rotating shift	-0.03	0.42	-0.03	0.54	-2.17	0.03	0.03	0.52	<0.01	1.00	0.03	0.53
Job satisfaction	Satisfied vs. dissatisfied	-11.42	<0.01	-5.01	<0.01	-4.26	<0.01	7.91	<0.01	-4.79	<0.01	-4.74	<0.01
	Neutral vs. dissatisfied	-4.21	<0.01	-1.90	0.01	-0.06	0.39	2.98	<0.01	-2.71	<0.01	-2.09	<0.01
Workplace bullying		0.29	<0.01	0.15	<0.01	0.05	0.39	0.02	0.65	0.15	<0.01	0.05	<0.01
Adjusted R^2^		0.50		0.31		0.07		0.25		0.34		0.33	
F (p)		80.85 (<0.01)		37.15 (<0.01)		12.44 (<0.01)		28.56 (<0.01)		41.65 (<0.01)		53.76 (<0.01)	

ProQoL: professional quality of life.

## Discussion

The present study is noteworthy in that it confirmed that workplace bullying had a significant effect on emotional exhaustion and depersonalization among the subdomains of burnout, on compassion fatigue among ProQoL, and on turnover intention. The mean score of workplace bullying obtained in this study was 39.29, which is similar to that in Yeun’s study [30] with general hospital nurses in South Korea. However, the degree of workplace bullying was found to be higher in the present study compared with studies with Japanese nurses (mean score 29.7) [31] and Canadian nurses (mean score 34.5) [32]. This could be due to a higher percentage of nurses who had less than 2 years of experience among the participants in the present study (38.3%). Their relatively low position within the nursing organization and their job performance reflecting a need for further skill could be the reason for their more frequent workplace bullying experiences. However, studies with American and Taiwanese nurses [33,34] showed higher scores on personal bullying, while this and another study with Korean nurses [35] showed higher scores on work-related bullying compared to personal or intimidation-related bullying. Considering the structure of nursing manpower in South Korea, with 5.2 nurses per 1,000 people, which is half of the average in OECD countries at 9.2 nurses per 1,000 people [36], it is likely that heavy workload, consequent pressure, and high level of conflict between staff may lead to higher workplace bullying, particularly work-related, in South Korea than in other countries.

The frequency of intimidation-related bullying was relatively low in this study, as was also in previous studies [37]. There is a possibility of over- or underestimation of workplace bullying resulting from cultural and linguistic differences in the measurement tool. Although the NAQ-R is used as a validated tool for measuring workplace bullying worldwide, it was developed based on the jobs and organizational culture of Western society [23]. Therefore, cultural and linguistic differences need to be considered. Further comparisons of workplace bullying between countries need to be conducted and analyzed using other validated tools.

In this study, nurses who had less than 5 years of experience, worked rotational shiftwork, and/or were dissatisfied with their job showed higher levels of workplace bullying, burnout, and turnover intention, and lower ProQoL. It was reported that nurses experience burnout due to exhaustion, constant stress, and helplessness resulting from exposure to rude behaviors in the workplace [9]. In particular, in a previous study with newly hired nurses, 29.5% of them were considering quitting their job [38]. Moreover, it has been reported that nurse administrators’ responses included taking charge, supporting staff, and doing nothing [39]. Another study found that when nurses complained of workplace bullying, nurse managers’ or institutions’ reactions involved ignoring the complaints or treating them as not important, or moving harassing nurses to other departments with fewer nurses [40]. Nursing administrators must recognize the problem of bullying. Continuous education is essential to identify workplace bullying and to properly deal with it by adopting preventive measures; thus, procedures and policies need to be developed. Nurses should be supported and encouraged by promoting communication, training in self-assertion, mentoring, cognitive rehearsal, and improving the organizational culture [41].

In a recent study, compassion satisfaction was significantly lower and burnout was higher when verbal and physical violence from coworkers were prevalent [28]. Moreover, a study with 5,000 nurses in Finland [42] found that most nurses who had experienced workplace bullying had turnover intention. When faced with bullying or lack of respect, some nurses may initially attempt to cope with it by trying to enhance their status and professionalism, but continued alienation could lead them to consider switching jobs [43]. Therefore, to tackle the burnout, reduced ProQoL, and turnover intention resulting from bullying experience, it is necessary to implement periodic and systematic monitoring, an active intervention system, and an institutional strategy for reporting bullying, holding employees accountable, and correcting unacceptable bullying behavior.

Workplace bullying among nurses is the most significant factor affecting their turnover intention, resulting in physical, emotional, and occupational devastation of individuals, as found in this study. Workplace bullying prevention guidelines for healthcare and social service workers were released in the United States [44], and workplace bullying and harassment prevention guidelines were published in Europe [45]. The World Health Organization has also published workplace bullying management guidelines [46]. Moreover, programs for managing the risk of workplace bullying in healthcare institutions have been developed [47]. In the United States, rapid response teams can take action against workplace violence [48]. The American Nurses Association [49] published a statement denouncing workplace bullying, and most medical institutes are implementing zero-tolerance policies to reduce disruptive behaviors. The concept of workplace bullying was noted in the revised Labor Standards Act from July 2019 in South Korea, and legislation addressing those who violate this law was implemented. When recognizing workplace bullying, employers are obliged to take appropriate action, such as protecting victims and disciplining offenders. However, there are no direct penalties for workplace bullying or clear guidelines in South Korea [50]. Securing adequate nursing labor and improving both the work environment and the nursing organizational culture are crucial to put an end to the continuous cycle in which shortage of nursing labor and a poor work environment result in work overload, bullying, burnout, and turnover.

In this study, the correlation between workplace bullying and decreased personal accomplishment was not statistically significant. According to a study by Leiter and Maslach [51], the core burnout domain directly affected by workplace bullying is emotional exhaustion, however, if bullying persists, it could lead to depersonalization and decreased personal accomplishment. In this study, workplace bullying also strongly affected emotional exhaustion (B = 0.29, p < 0.01, adjusted R^2^ = 0.50). Therefore, further studies are needed to further investigate the effect of workplace bullying on each domain of burnout.

This study has some limitations. First, convenience sampling was used to select nurses working at general hospitals in limited regions; thus, the results cannot be generalized to all nurses in South Korea. In the future, probability sampling should be expanded to medical institutes of all regions, including acute care hospitals and long-term nursing care centers. Second, owing to the cross-sectional design of the study, the causality between variables cannot be accurately inferred. Consistent replication studies on the effect of workplace bullying on nurses, nursing organizations, and patient care outcomes will be needed. Despite such limitations, this study is significant in that it comprehensively examined the association of workplace bullying with burnout, ProQoL, and turnover intention. We hope that guidelines for the identification, prevention, and management of workplace bullying as well as education and intervention programs will be established. To achieve this, conversations with and counseling of individual employees are required, as well as efforts from leaders who can actively encourage employee participation in the process of developing interventions.

## Conclusion

Workplace bullying in the nursing profession should not be ignored or overlooked. The role of nursing leadership is critical to reduce workplace bullying among nurses and create a work environment that is safe and healthy. More proactive mediations and strategies from various angles based on available data are needed. Therefore, in order to tackle bullying, interventions at the organizational level need to be established whereby nursing administrators or experts can protect nurses who experience workplace bullying.

## Supporting information

S1 Raw Data (SAV)
