# Correction: Association between workplace bullying and burnout, professional quality of life, and turnover intention among clinical nurses

**DOI:** 10.1371/journal.pone.0228124

**Published:** 2020-01-16

**Authors:** Yujeong Kim, Eunmi Lee, Haeyoung Lee

There is an error in affiliation 3 for author Haeyoung Lee. The correct affiliation 3 is: Red Cross College of Nursing, Chung-Ang University, Seoul, Republic of Korea.
